# New Cell Adhesion Molecules in Human Ischemic Cardiomyopathy. *PCDHGA3* Implications in Decreased Stroke Volume and Ventricular Dysfunction

**DOI:** 10.1371/journal.pone.0160168

**Published:** 2016-07-29

**Authors:** Ana Ortega, Carolina Gil-Cayuela, Estefanía Tarazón, María García-Manzanares, José Anastasio Montero, Juan Cinca, Manuel Portolés, Miguel Rivera, Esther Roselló-Lletí

**Affiliations:** 1 Cardiocirculatory Unit, The Health Research Institute La Fe, Valencia, Spain; 2 Cardiovascular Surgery Service, University and Polytechnic La Fe Hospital, Valencia, Spain; 3 Cardiology Service of Santa Creu i Sant Pau Hospital, Barcelona, Spain; Mount Sinai School of Medicine, UNITED STATES

## Abstract

**Background:**

Intercalated disks are unique structures in cardiac tissue, in which adherens junctions, desmosomes, and GAP junctions co-localize, thereby facilitating cardiac muscle contraction and function. Protocadherins are involved in these junctions; however, their role in heart physiology is poorly understood. We aimed to analyze the transcriptomic profile of adhesion molecules in patients with ischemic cardiomyopathy (ICM) and relate the changes uncovered with the hemodynamic alterations and functional depression observed in these patients.

**Methods and Results:**

Twenty-three left ventricular tissue samples from patients diagnosed with ICM (n = 13) undergoing heart transplantation and control donors (CNT, n = 10) were analyzed using RNA sequencing. Forty-two cell adhesion genes involved in cellular junctions were differentially expressed in ICM myocardium. Notably, the levels of protocadherin *PCDHGA3* were related with the stroke volume (*r* = –0.826, *P* = 0.003), ejection fraction (*r* = –0.793, *P* = 0.004) and left ventricular end systolic and diastolic diameters (*r* = 0.867, *P* = 0.001; *r* = 0.781, *P* = 0.005, respectively).

**Conclusions:**

Our results support the importance of intercalated disks molecular alterations, closely involved in the contractile function, highlighting its crucial significance and showing gene expression changes not previously described. Specifically, altered *PCDHGA3* gene expression was strongly associated with reduced stroke volume and ventricular dysfunction in ICM, suggesting a relevant role in hemodynamic perturbations and cardiac performance for this unexplored protocadherin.

## Introduction

Ischemic cardiomyopathy (ICM) leads to heart failure (HF) and is one of the major diseases that threaten human health, with high rates of morbidity and mortality [1]. HF is characterized by significant changes in the myocardium such as modification of organ structure and tissue organization, thereby resulting in adverse remodeling to overcome depressed cardiac function [2–5]. Among them, cell adhesion molecules (CAMs) are relevant structural players for this pathology [6]. One of the most crucial structures unique in cardiac tissue is the intercalated disc (ID), which is composed of three different cell adhesion types: gap junctions (GJ), desmosomes, and adherens junctions (AJ) [7]. The ID is a complex region in the cell, essential for electrical and mechanical signal transduction between cells and, hence, crucial for the growth and functioning of the heart. Specifically, ID has been shown to be important for maintaining the structural integrity and synchronized contraction of the cardiac tissue [8].

Connexins are specific structural proteins of GJ [9], while the superfamily of cadherins (calcium-dependent adhesion molecules) are present at desmosomes (desmogleins and desmocollins) and AJs (classical cadherins and protocadherins) [10, 11]. Previous studies have reported the associations of desmosomal and classical cadherins with cardiovascular diseases [12–14]. Protocadherins are a family of adhesion molecules, which differ from cadherins in their structure and function [15]. Protocadherins have been shown to be associated with cancer and neurological disorders [16, 17]. However, their implications in HF or other cardiovascular diseases remain to be elucidated.

We hypothesize that in HF patients, there may exist important changes in adhesion molecules influencing the impaired cardiac function. To test this hypothesis, we investigated, using RNA sequencing (RNA-seq), differentially expressed adhesion-related genes in ICM patients compared to the control (CNT) group and related them with cardiac hemodynamic perturbations and left ventricular (LV) dysfunction.

## Materials and Methods

### Source of tissue samples

LV samples were obtained from 13 ICM patients undergoing cardiac transplantation and 10 non-diseased donor hearts were used as CNT samples. To improve the numerical base to a higher number of patients for protein analysis, we increased the ICM samples to 21. The clinical history, ECG, doppler echocardiography, hemodynamic studies, and coronary angiography data were available on patients. Patients with primary valvular disease were excluded from the study. All subjects were functionally classified based on the New York Heart Association (NYHA) criteria and received medical treatment in accordance with the guidelines of the European Society of Cardiology [2]. Table 1 shows the clinical characteristics of patients included in this study.

**Table 1:** Clinical characteristics of ischemic cardiomyopathy (ICM) patients.

	ICM (n = 13)	ICM (n = 21)
	RNA sequencing	Western blot
Age (years)	54±7	55±7
Gender male (%)	100	91
NYHA class	III-IV	III-IV
BMI (kg/m^2^)	26±4	26±4
Haemoglobin (mg/dL)	14±3	14±2
Haematocrit (%)	41±6	41±5
Total cholesterol (mg/dL)	162±41	162±42
Prior hypertension (%)	30	32
Prior smoking (%)	84	90
Diabetes mellitus (%)	38	47
EF (%)	24±4	23±6
LVESD (mm)	55±7	58±9
LVEDD (mm)	64±7	66±9

ICM, ischemic cardiomyopathy; NYHA, New York Heart Association; BMI, body mass index; EF, ejection fraction; LVESD, left ventricular end-systolic diameter; LVEDD, left ventricular end-diastolic diameter.

LV samples were collected from near the apex of the left ventricle and maintained in 0.9% NaCl at 4°C for a maximum of 4.4±3 h after the coronary circulation loss, and then stored at –80°C until RNA extraction and protein determination. The appropriate handling and rapid sample collection and storage by our on call (24 h) team, lead to the collection of these high quality samples (RNA Integrity Number (RIN) > 9 for all samples). The sample’s handling was carried out equally in both groups.

The CNT hearts were initially considered for cardiac transplantation donation, however, were subsequently deemed unsuitable owing to the incompatibility in blood type or heart size. The causes of death were either cerebrovascular or motor vehicle accidents. All hearts had normal LV function and no history of myocardial disease at the time of transplantation. Owing to our national data protection law (Organic Law on Data Protection 15/1999), we only have access to the knowledge of age and sex characteristics, being impossible for us to provide other data of this group.

The project was approved by the Ethics Committee of University and Polytechnic Hospital La Fe and was conformed in accordance with the principles outlined in the Declaration of Helsinki [18]. All heart samples were obtained with written informed consent of patients.

### RNA isolation

TRIzol^®^ agent was used to homogenize tissue samples in TissueLyser LT (Qiagen; UK). RNA was extracted using the PureLink^™^ Kit (Ambion Life Technologies; CA, USA), following the manufacturer’s recommendations. The RNA concentration was measured on the Nanodrop 1000 spectrophotometer (Thermo Fisher Scientific; UK), and the purity and integrity of RNA samples were measured using the microfluidics-based platform 2100 Bioanalyzer with the RNA 6000 Nano LabChip Kit (Agilent Technologies; Spain). All RNA samples displayed a 260/280 absorbance ratio ≥2.0 and reached a minimal RIN ≥9.

### RNA-seq analysis

The poly(A)-RNA samples were isolated from 25 μg of total RNA using the MicroPoly(A) Purist Kit (Ambion; USA). The SOLiD 5500 XL platform (Life Technologies; CA, USA) was used for sequencing whole transcriptome libraries generated from total poly(A)-RNA samples, following the manufacturer’s instructions. No RNA-spike was used in controls. Amplified cDNA quality was analyzed by the Bioanalyzer 2100 DNA 1000 Kit (Agilent Technologies; Spain) and quantified by the Qubit 2.0 Fluorometer (Invitrogen; UK). The whole transcriptome libraries were used for making SOLiD-templated beads, following the SOLiD Templated Bead Preparation guidelines. The bead quality was assessed based on the workflow analysis parameters. The samples were sequenced using the 50625 paired-end protocol generating 75 nt + 35 nt (paired-end) + 5 nt (barcode) sequences. Quality data were measured using SOLiD Experimental Tracking Software parameters.

### RNA-seq data computational analysis

The initial whole transcriptome paired-end reads obtained from sequencing were mapped against the latest version of the human genome (version GRchr37/hg19) by using the Life Technologies mapping algorithm (http://www.lifetechnologies.com/; version 1.3). The aligned records were reported in BAM/SAM format [19]. The Picard Tool (http://picard.sourceforge.net/; version 1.83) was used to eliminate insufficient quality reads (Phred score <10). Subsequently, gene predictions were estimated by the Cufflinks method [20] and the expression levels were calculated by using HTSeq software [21]. This method eliminates the multi-mapped reads, only the unique reads are considered for gene expression estimation. The differential expression analysis between conditions was assessed by the edgeR method (version 3.2.4) [22]. This method relies on different normalization process based on the depth of global samples, the CG composition and the gene length. Moreover, is based on a Poisson model that estimates the variance of the RNA-seq data for differential expression. Finally, we selected genes showing differential expression at a significance threshold of *P* < 0.05. The data presented in this manuscript have been deposited in NCBI’s Gene Expression Omnibus (GEO) [23] and are accessible through GEO Series accession number GSE55296 (http://www.ncbi.nlm.nih.gov/geo/query/acc.cgi?acc=GSE55296).

### Homogenization of samples and protein determination

Twenty-five milligrams of frozen left ventricle were transferred into Lysing Matrix D tubes designed for the FastPrep-24 homogenizer (MP Biomedicals, USA) in a total protein extraction buffer (2% SDS, 10 mM EDTA, 6 mM Tris–HCl, pH 7.4) with protease inhibitors (25 μg/mL aprotinin and 10 μg/mL leupeptin). The homogenates were centrifuged and supernatant aliquoted. The protein content of the aliquot was determined using Peterson’s modification of the micro Lowry method with bovine serum albumin (BSA) as the standard.

### Polyacrylamide gel electrophoresis and western blot analysis

Protein samples for the detection of PCDHGA3 were separated using Bis-Tris Midi gel electrophoresis with 4–12% polyacrylamide under non-reducing conditions. After electrophoresis, the proteins were transferred from the gel to a PVDF membrane using the iBlot Dry Blotting System (Invitrogen Ltd, UK) for western blot analysis. The membranes were blocked overnight at 4°C with 1% BSA in Tris buffer solution containing 0.05% Tween 20 and, after blocking, were incubated for 2 h with primary antibody in the same buffer. The following antibodies were used: anti-PCDHGA3 goat polyclonal, from Santa Cruz Biotechnology (sc-109811; 1/100), and anti-GAPDH (loading control) mouse monoclonal, from Abcam (ab9484; 1/1000). The bands were visualized using an acid phosphatase-conjugated secondary antibody and nitro blue tetrazolium/5-bromo-4-chloro-3-indolyl phosphate (NBT/BCIP, Sigma-Aldrich, St. Louis, USA) substrate system. Finally, the membranes were digitalized using an image analyzer (DNR Bio-Imagining Systems, Israel) and quantified with the GelQuant Pro (v. 12.2) program.

### Gene functional characterization

The functional enrichment of differentially expressed genes was based on hypergeometric test using ToppGene suite [24].We selected the differentially expressed genes from ICM patients with ≥1.3–fold and *P* < 0.05 by using Bonferroni correction. Next, the most significant functional categories altered in ICM patients were represented.

### Statistical methods

Data were expressed as the mean ± standard deviation for continuous variables and as percentage values for discrete variables. The Kolmogorov–Smirnov test was applied for analyzing the data distribution. Clinical characteristics of patients were compared by using Student’s t-test for continuous variables and Fisher’s exact test for discrete variables. Significant mean differences between groups with a normal distribution were analyzed by using the Student’s *t*-test, whereas the non-parametric Mann–Whitney *U* test was performed for comparisons between data that were non-normally distributed. The *ITGAM* and *VCAM* mRNA levels exhibited a non-normal distribution and were log transformed (and proved to be normalized) before parametric correlation analysis. Finally, Pearson’s correlation coefficients were calculated to determine the relationships among variables. Multivariate linear regression analysis was achieved using ejection fraction (EF) and stroke volume as dependent variables and age, gender, and LV diameters as independent variables. Best model discrimination was based on the principle of least mean square and greatest *r*^*2*^. *P* < 0.05 was considered statistically significant. All statistical analyses were performed using the SPSS software (version 20.0) for Windows (IBM SPSS Inc.; Chicago. IL, USA).

## Results

### Clinical characteristics of patients

We analyzed a total of 21 LV tissue samples from ICM patients undergoing heart transplantation and 10 CNT hearts. For RNA-seq analysis (n = 13) the ICM patients were all men, with a mean age of 54 ± 7 years. We increased the sample size to up to 21 LV tissue samples for western blot analysis, with 91% patients being men of mean age 55 ± 7 years. The patients were previously diagnosed with comorbidities including hypertension (30% and 32%, respectively) and diabetes mellitus (38% and 47%, respectively). All patients had suffered bundle branch block, observed in their electrocardiographic tracing, including LBBB, RBBB, completes and incompletes blocks. And 15% were treated with a CRT therapy before cardiac transplantation. Table 1 shows the clinical characteristics of the patients included in the study. The CNT group was comprised mainly of men (80%), with a mean age of 47 ± 16 years.

### Gene expression analysis by RNA-Seq and enrichment of functional categories

To investigate the alterations accompanying human ICM pathology, we performed a large-scale expression analysis in 23 LV samples (ICM, n = 13; CNT, n = 10) by using RNA-seq technology. We identified 1712 differentially expressed genes between the ICM and CNT groups (≥1.3–fold, *P* < 0.05), among which 815 were up-regulated and 897 were down-regulated.

**Fig 1:**
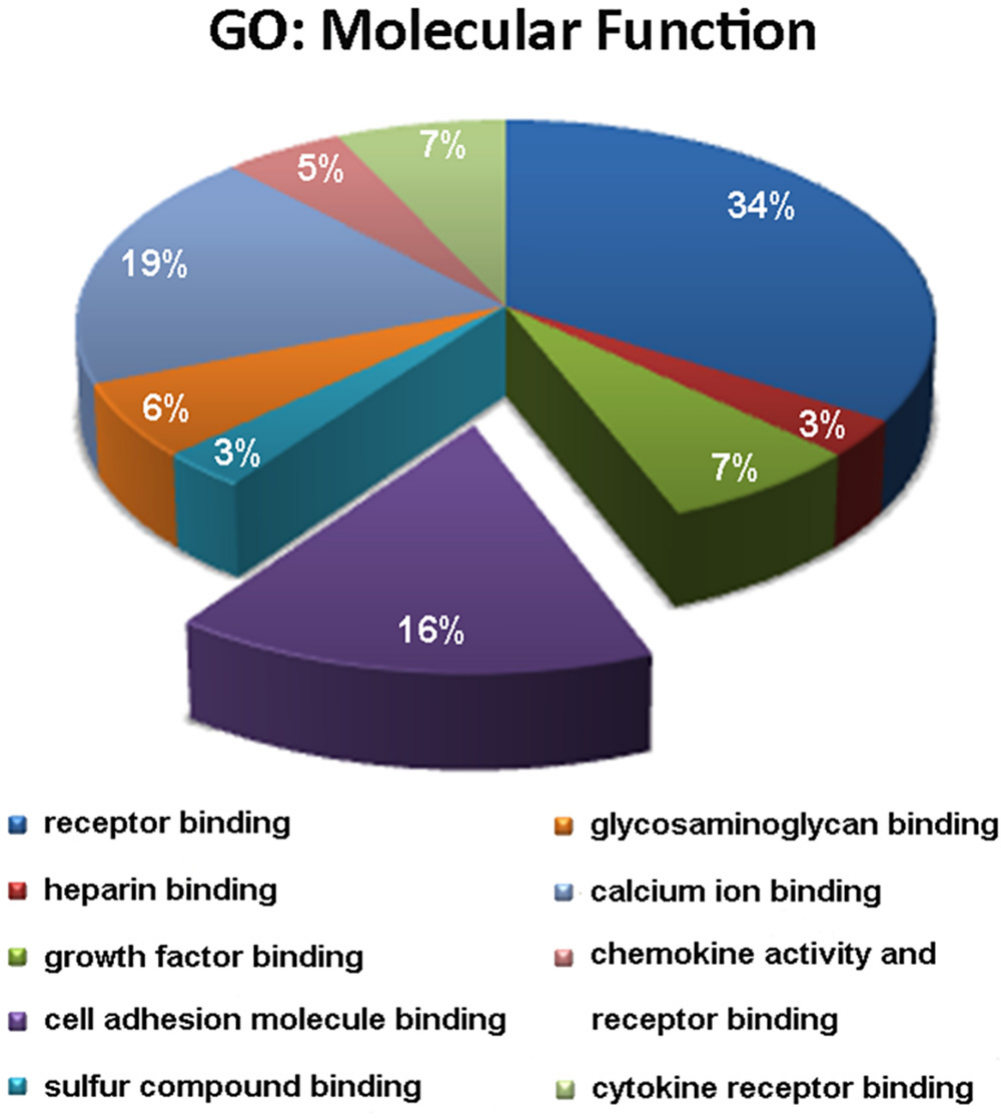
Functional enrichment representation showing that cell adhesion is the third most altered category in ICM pathology. ToppGene results in terms of Gene ontology (GO) were obtained based on Molecular Function and were corrected using Bonferroni method. The diagram includes the main categories in which differentially expressed genes are classified. ICM, ischemic cardiomyopathy.

We used the ToppGene tool to determine the Gene Ontology (GO) categories encompassing the differentially expressed genes of ICM samples. We analyzed the GO terms in the “Molecular Function” category to ascertain the main biological functions in which the deregulated genes are implicated. The third most relevant functional category was related to cell adhesion, representing 16% of the total categories (Fig 1).

**Fig 2:**
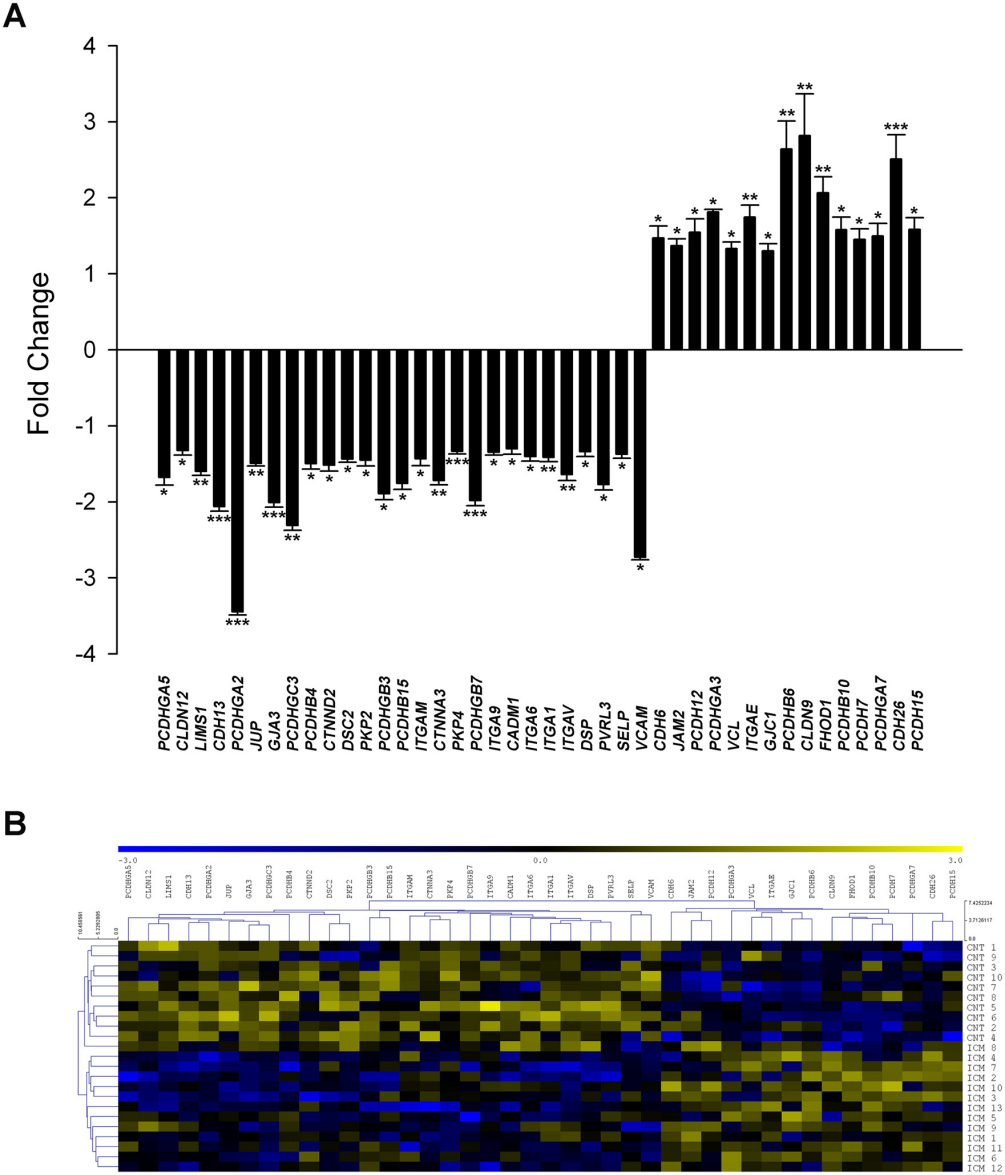
Differential gene expression profiles of cell adhesion molecules in ICM patients displaying 42 altered genes. **(A)**. RNA-sequencing results showing mRNA expression levels of cell adhesion genes. The values of the CNT group are set to 1. The data are expressed as mean ± SEM for the relative mRNA expression levels. **(B)**. Heat map with hierarchical clustering of the transcriptomic analysis. Columns: genes; rows: samples. The relative expression level of each gene is indicated by the colour bar: blue, lowest; yellow, highest. ICM, ischemic cardiomyopathy; CNT, control. *P < 0.05, **P < 0.01, ***P < 0.001 *vs* CNT group.

Among the altered genes, we focused on cell adhesion related genes, thereby finding 42 differentially expressed genes, of which 27 were down-regulated and 15 up-regulated (Fig 2A). We performed a hierarchical clustering and heat map analysis to better visualize the altered expression of genes in the cell adhesion category, which clearly identified the ICM and CNT groups in two different expression patterns (Fig 2B). The differentially expressed genes belonging to CAMs found in RNA-seq analysis are shown in Table 2, and the relationships found between them are summarized in S1 Table.

**Table 2:** Differentially expressed genes of adhesion molecules in ICM patients.

Gene symbol	Description	Fold Change	*P*-value
*CADM1*	Cell adhesion molecule 1	-1.30	0.037
*CDH6*	Cadherin 6, type 2, K-cadherin	1.47	0.042
*CDH13*	Cadherin 13, H-cadherin	-2.06	6.1 x 10^−4^
*CDH26*	Cadherin-like protein 26	2.51	5.8 x 10^−4^
*CLDN9*	Claudin 9	2.82	0.008
*CLDN12*	Claudin 12	-1.32	0.025
*CTNNA3*	Catenin alpha 3	-1.71	0.004
*CTNND2*	Catenin delta 2	-1.52	0.024
*DSC2*	Desmocollin 2	-1.44	0.041
*DSP*	Desmoplakin	-1.34	0.011
*FHOD1*	Formin homology 2 domain containing 1	2.07	0.001
*GJA3*	GAP junction alpha protein 3, connexin 46	-2.01	7.1 x 10^−4^
*GJC1*	GAP junction gamma protein 1, connexin 45	1.30	0.010
*ITGA1*	Integrin, alpha 1	-1.42	0.004
*ITGA6*	Integrin, alpha 6	-1.40	0.028
*ITGA9*	Integrin, alpha 9	-1.34	0.047
*ITGAE*	Integrin, alpha E	1.75	0.003
*ITGAM*	Integrin, alpha M	-1.43	0.031
*ITGAV*	Integrin, alpha V	-1.64	0.005
*JAM2*	Junctional adhesion molecule B	1.36	0.018
*JUP*	Junction plakoglobin	-1.49	0.006
*LIMS1*	LIM and senescent cell antigen-like-containing domain protein 1	-1.60	0.001
*PCDH7*	Protocadherin 7	1.45	0.048
*PCDH12*	Protocadherin 12	1.55	0.033
*PCDH15*	Protocadherin 15	1.59	0.031
*PCDHB4*	Protocadherin beta 4	-1.50	0.025
*PCDHB6*	Protocadherin beta 6	2.64	0.001
*PCDHB10*	Protocadherin beta 10	1.58	0.025
*PCDHB15*	Protocadherin beta 15	-1.76	0.042
*PCDHGA2*	Protocadherin gamma subfamily A, 2	-3.45	4.1 x 10^−4^
*PCDHGA3*	Protocadherin gamma subfamily A, 3	1.81	0.035
*PCDHGA5*	Protocadherin gamma subfamily A, 5	-1.68	0.031
*PCDHGA7*	Protocadherin gamma subfamily A, 7	1.50	0.020
*PCDHGB3*	Protocadherin gamma subfamily B, 3	-1.89	0.039
*PCDHGB7*	Protocadherin gamma subfamily B, 7	-1.98	6.5 x 10^−4^
*PCDHGC3*	Protocadherin gamma subfamily C, 3	-2.31	0.006
*PKP2*	Plakophilin 2	-1.46	0.044
*PKP4*	Plakophilin 4	-1.34	1.0 x 10^−4^
*PVRL3*	Nectin 3	-1.77	0.005
*SELP*	Selectin-P	-1.38	0.017
*VCL*	Vinculin	1.33	0.039
*VCAM1*	Vascular cell adhesion molecule 1	-2.73	0.011

### Relationships between differentially expressed genes and stroke volume and ventricular function

**Fig 3:**
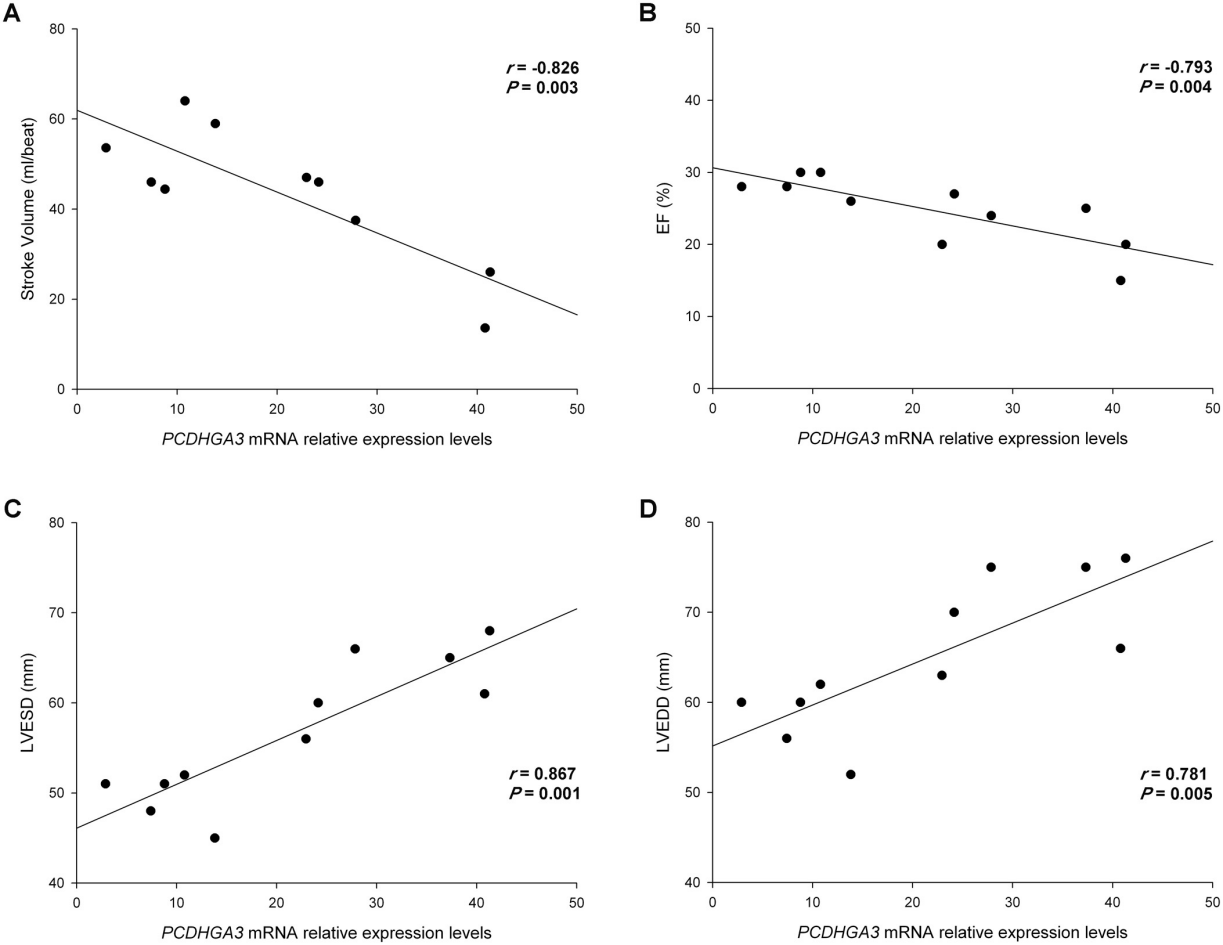
Scatter plots showing the correlations between *PCDHGA3* with left ventricular dysfunction and stroke volume in ICM patients. **(A)** Stroke volume. **(B)** Ejection fraction (EF). **(C)** Left ventricular end-systolic diameter (LVESD). **(D)** Left ventricular end-diastolic diameter (LVEDD). ICM, ischemic cardiomyopathy.

We investigated whether there was any relationship between the altered genes and hemodynamic and echocardiographic parameters of ICM patients. We had completely available the hemodynamic parameters data of 10 ICM patients and the LV function parameters of 11 ICM patients. We related the expression of all altered genes with these parameters and we found that only the protocadherin *PCDHGA3* showed relationships with both hemodynamic and functional parameters. These results are summarized in S2 Table. *PCDHGA3* was inversely related with the stroke volume (*r* = –0.826, *P* = 0.003; Fig 3A). When adjusting the model for age, gender and LV diameters, the significance level was maintained (*r*^*2*^ = 0.684, *P* = 0.040). We also found an inverse relationship between this protocadherin with EF (*r* = –0.793, *P* = 0.004; Fig 3B) and was directly related to the LV end-systolic diameter (LVESD) and LV end-diastolic diameter (LVEDD) (*r* = 0.867, *P* = 0.001; *r* = 0.781, *P* = 0.005, respectively) (Fig 3C and 3D). The adjustment of model for age, gender, and LV diameters maintained the relationship between *PCDHGA3* expression and EF, obtaining an *r*^*2*^ = 0.976 and *P* = 0.001.

### Western blot analysis

**Fig 4:**
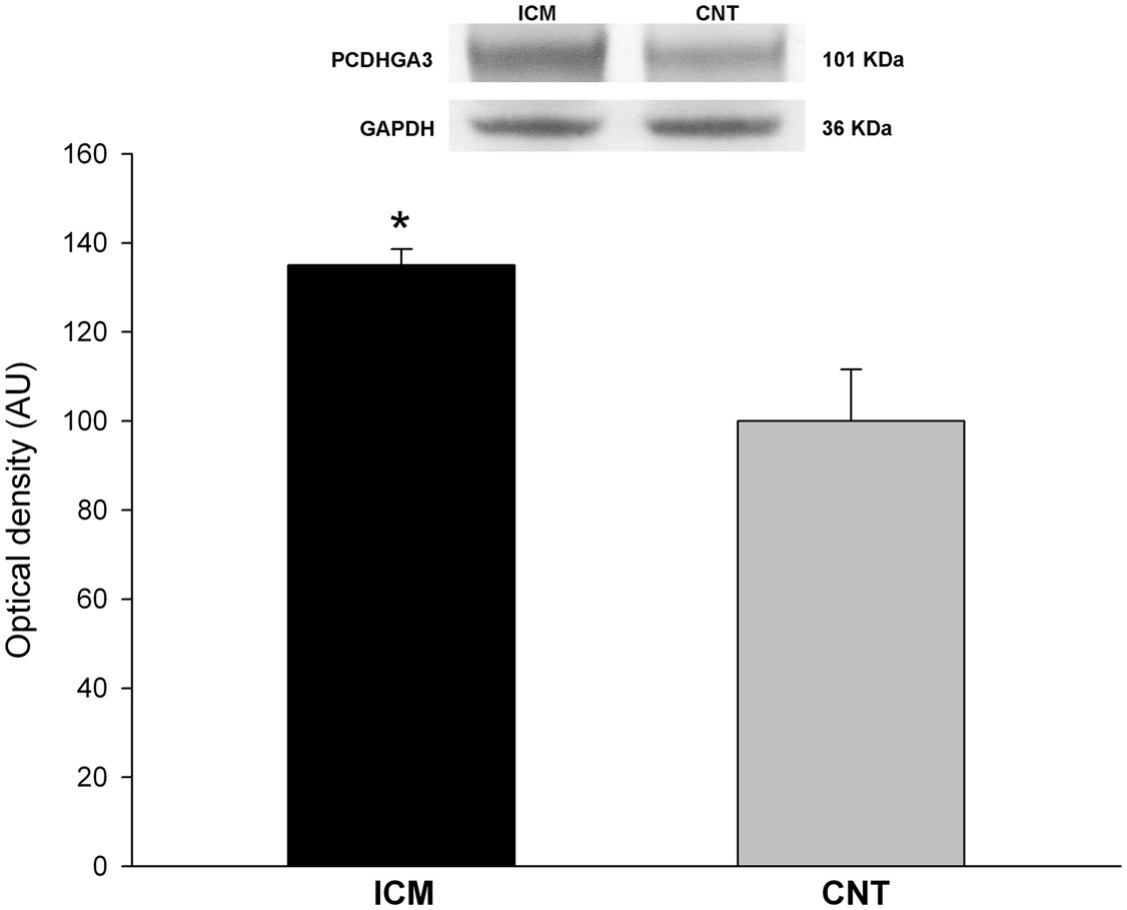
Increased protein levels of PCDHGA3 in ICM patients. The values of the CNT group, previously normalized to GAPDH, were set to 100. The data are expressed as mean ± SEM in optical density arbitrary units (AU). *P < 0.001, *vs* the CNT group. ICM, ischemic cardiomyopathy; CNT, control.

We analyzed the protein levels of PCDHGA3 due to its relationship with cardiac hemodynamic condition and ventricular dysfunction. We found that the levels of PCDHGA3 were in accordance with the previously measured mRNA levels (135 ± 16 *vs* 100 ± 28 arbitrary units (AU), *P* < 0.001) (Fig 4).

## Discussion

In the present study, we performed a transcriptomic analysis of HF patients with ischemic origin through RNA-seq technique, identifying 42 deregulated genes related to CAMs. This category, evidenced by the gene enrichment analysis, emerged as a relevant player in ICM, being the third most altered. CAMs mediate junction formation between cells and with extracellular matrix, thus participating in tissue structure organization and being important modulators of signal transduction. In ICM patients, we found alterations in the expression of genes associated with all types of junctions. As described above, most of these cell adhesions constitute a specialized connection between cardiomyocytes, the ID [8]. Since the conventional functions of this structure include providing mechanical attachment in myocardium and allowing signal communication between cardiomyocytes, our results could help to identify new molecules involved in human HF progression. Together, our analysis uncovers differential expression patterns of genes not previously implicated in ICM pathology.

We found some representative genes of desmosome structure altered, including *DSP*, *JUP*, *PKP2/4* and *DSC2*, genes that express the junction proteins desmoplakin, plakoglobin, plakophilin and desmocollin, respectively, which perform the ID structure together with GJ and AJ. Our data are consistent with the previous studies that have related decrease in signaling at the ID and loss of function mutations of these adhesion molecules to HF and arrhythmogenic right ventricular cardiomyopathy (ARVC) [12, 25–28]. Our results revealed gene expression down-regulations of desmosomal components in ICM patients, and also important relationships with altered protocadherins and integrins that could be influencing the ID structure and function in HF, as demonstrated by several reports that suggest a structural disorganization of ID in this syndrome [6, 29].

We also identified genes differentially expressed in ICM encoding GJ molecules, such as *GJC1* (connexin 45) and *GJA3* (connexin 46). Connexins regulate action potential transfer between cardiomyocytes and loss of function of these proteins has been previously associated with HF [30]. Similar to desmosomal genes, connexins also have a link with HF and ARVC and reduced connexin expression in these patients leads to an increase in ventricular arrhythmias and sudden death [31, 32]. Specifically, connexin-43 protein has been shown to be reduced in experimental models and in human HF [33, 34], however few studies analyze its gene expression in patients and show contradictory results [35, 36]. We found no significant differences in the connexin-43 coding gene *GJA1* in ICM patients, thereby suggesting that the reported reduction of its expression could be due to post-translational modifications. Of the connexin altered genes in our study, only *GJA3* has been previously reported to play a role in HF affecting cardiac conduction [37], being *GJC1* a not previously described gene in ICM pathology. Moreover, we found significant relationships between desmosomal and connexin genes, supporting the finding that there is a link between them [38], and contributing also to the disorganization of ID in myocytes.

We observed that the AJ (cadherins, catenins and protocadherins) were the most altered genes. A total of 3 cadherins, 2 catenins and 14 protocadherins were differentially expressed in ICM patients. Cadherins constitute a big family of calcium-dependent cell-cell adhesive proteins, and their expression is particularly important in the heart, which is under constant mechanical load [39]. Moreover, we found good associations between the members of these families, showing the existence of crosstalk between them, and suggesting that changes in the expression of one adhesion gene could influence the levels of others. While cadherins were mostly up-regulated, the opposite was observed for catenins, and protocadherin sub-family expression was found to be altered in both directions. Protocadherins have been mainly studied in the context of the nervous system, where they participate in the establishment of specific neuronal connectivity and mediate intercellular adhesions and signaling [40]. Interestingly, we show that *PCDHGA3* has a good and inverse relationship with stroke volume and cardiac function, indicating that high expression levels are related with reduced stroke volume and ventricular dysfunction. Consistently, we also found that the protein levels of this protocadherin showed the same tendency of expression in ICM patients. The specific function of *PCDHGA3* in the heart has not been established yet, and only one study has described its role in disease [41]. Few evidences are known regarding its function in other tissues, but it seem that participates in shaping intercellular membranes [42]. Among the other altered genes, it was the only one related to both functional and hemodynamic status of ICM patients at a great significance level. These parameters reflect the alterations in cardiac contractility and remodeling processes that occur in ICM, which at molecular level, are translated into activation of different pathways such as fibrosis and apoptosis. The described function for protocadherins in the maintenance of cell adhesion interactions is critical for contraction coupling of cardiomyocytes. Maybe, although further studies need to be done, PCDHGA3 could play also an important role in other of these functions related to the ICM progression, apart from the physical interactions of cell adhesions, which can be both contributing to these clinical status relationships, as evidenced by the distinct role proposed for the protocadherin PCDH1. This molecule has been shown to have a role in the regulation of TGF-β in epithelial cells, which is involved in many cellular pathways such as transcription activation of extracellular matrix genes or apoptosis, process highly activated in ICM pathology [43]. There are also evidences of relationships between alterations of cadherins expression and cardiac function [44], and about the involvement of cadherins in extracellular matrix remodeling necessary for fibrosis progression [45]. Taking into account these different functions for cadherin superfamily, we purpose a link between molecular alterations of *PCDHGA3* and heart dysfunction, in which the high levels found of this molecule may be increasing cellular adhesions or regulating distinct processes related to heart function in a potential attempt to restore the cardiac contractility and elasticity in ischemic hearts, evidenced by the strong relationships found with stroke volume and heart dysfunction. In the light of these findings, our results could provide a basis for studying the implications of protocadherin subfamily, and specifically *PCDHGA3* in cardiac hemodynamic status and LV performance of ICM patients.

### Study limitations

As with any study using human samples, the inherent variability and the differential effect of administered medications could affect the mRNA levels in our patients. However, it is crucial to emphasize the importance of having carried out this study in a significant number of ICM samples from explanted human hearts undergoing cardiac transplantation and CNT donors, making our results applicable for ICM population.

### Conclusion

Our results support the importance of IDs alterations closely involved in the contractile function, highlighting its crucial significance and showing gene expression changes that have not been previously described. Specifically, altered *PCDHGA3* gene expression was strongly associated with cardiac stroke volume and ventricular dysfunction in ICM, showing its relevance in cardiac hemodynamic perturbations and LV performance for this unexplored protocadherin.

## Supporting Information

S1 Table Relationships between differentially expressed cell adhesion genes in ICM patients. (DOCX)

S2 Table Relationships between differentially expressed cell adhesion genes and hemodynamic and LV function parameters of ICM patients. (DOCX)
