# Supplementary material for: New Cell Adhesion Molecules in Human Ischemic Cardiomyopathy. PCDHGA3 Implications in Decreased Stroke Volume and Ventricular Dysfunction
Source: PLoS One. 2016 Jul 29;11(7):e0160168. doi: 10.1371/journal.pone.0160168 (PMC4966940; doi:10.1371/journal.pone.0160168)
Supplement: S1 Table — (DOCX) [file pone.0160168.s001.docx]

**S1 Table. Relationships between differentially expressed cell adhesion genes in ICM patients.**

| **Genes** | **Pearson correlation** | ***P*-value** |
| --- | --- | --- |
| *CDH6 – VCL* | -0.575 | 0.040 |
| *CDH6 – PCDH7* | 0.656 | 0.021 |
| *CDH13 – CDH26* | -0.675 | 0.016 |
| *CDH13 – PCDHGA5* | 0.715 | 0.009 |
| *CDH13 – LIMS1* | 0.668 | 0.018 |
| *CDH13 – ITGA6* | 0.599 | 0.039 |
| *CDH13 – JAM2* | 0.667 | 0.018 |
| *CDH26 – ITGAM* | 0.626 | 0.030 |
| *CDH26 – PCDH15* | 0.632 | 0.020 |
| *CDH26 – PCDHGA5* | -0.664 | 0.013 |
| *CDH26 – PVRL3* | -0.619 | 0.042 |
| *CLDN9 – DSP* | -0.630 | 0.028 |
| *CLDN9 – JUP* | -0.716 | 0.009 |
| *CLDN9 – PCDHB4* | -0.705 | 0.015 |
| *CLDN12 – PCDH7* | -0.611 | 0.035 |
| *CLDN12 – PCDHGA5* | 0.702 | 0.007 |
| *CLDN12 – SELP* | -0.566 | 0.044 |
| *CLDN12 – LIMS1* | 0.840 | 0.001 |
| *CLDN12 – ITGAV* | 0.583 | 0.047 |
| *CLDN12 – FHOD1* | -0.559 | 0.047 |
| *CADM1 – DSP* | 0.762 | 0.002 |
| *CADM1 – PCDHGA7* | -0.622 | 0.041 |
| *CADM1 – PCDHGC3* | 0.676 | 0.032 |
| *CADM1 – PKP2* | 0.669 | 0.012 |
| *CADM1 – GJA3* | 0.616 | 0.033 |
| *CTNNA3– FHOD1* | 0.651 | 0.022 |
| *DSC2– ITGAV* | 0.601 | 0.039 |
| *DSP – CLDN9* | -0.630 | 0.028 |
| *DSP – JAM2* | 0.676 | 0.011 |
| *DSP – PCDH12* | 0.714 | 0.009 |
| *DSP – PCDH17* | 0.704 | 0.007 |
| *DSP – PKP2* | 0.752 | 0.003 |
| *DSP – PCDHGC3* | 0.674 | 0.033 |
| *DSP – PCDHGA7* | -0.700 | 0.016 |
| *DSP – VCL* | -0.649 | 0.016 |
| *DSP – LIMS1* | 0.587 | 0.035 |
| *DSP – ITGA1* | 0.590 | 0.034 |
| *DSP – ITGA6* | 0.825 | 0.001 |
| *DSP – ITGAV* | 0.741 | 0.006 |
| *DSP – FHOD1* | -0.680 | 0.010 |
| *GJA3 – ITGAV* | 0.678 | 0.022 |
| *GJA3 – JUP* | 0.660 | 0.019 |
| *GJA3 – PKP2* | 0.685 | 0.014 |
| *GJA3 – PCDH7* | -0.605 | 0.049 |
| *GJA3 – PCDHGA5* | 0.644 | 0.024 |
| *GJA3 – PCDHB10* | -0.702 | 0.024 |
| *GJA3 – PCDHGC3* | 0.771 | 0.009 |
| *JUP – PCDHGA5* | 0.602 | 0.029 |
| *JUP – PCDHB4* | 0.815 | 0.001 |
| *JUP – PCDHGC3* | 0.668 | 0.035 |
| *JUP – LIMS1* | 0.587 | 0.035 |
| *JUP – ITGA9* | -0.639 | 0.025 |
| *PKP2 – PCDHGA7* | -0.648 | 0.031 |
| *PKP2 – SELP* | -0.731 | 0.005 |
| *PKP2 – LIMS1* | 0.648 | 0.017 |
| *PKP2 – ITGAV* | 0.887 | 0.001 |
| *PKP2 – ITGA6* | 0.723 | 0.005 |
| *PKP2 – FHOD1* | -0.643 | 0.018 |
| *PKP4 – PCDHB4* | -0.711 | 0.010 |
| *PCDH12– ITGA1* | 0.649 | 0.022 |
| *PCDH12– ITGA6* | 0.910 | 0.001 |
| *PCDH12– ITGA9* | 0.664 | 0.023 |
| *PCDH15– FHOD1* | 0.556 | 0.048 |
| *PCDHGA3– ITGB7* | 0.686 | 0.042 |
| *PCDHGB3 – PCDHB6* | -0.597 | 0.041 |
| *PCDHGB3 – ITGB7* | 0.752 | 0.012 |
| *PCDHGA5 – LIMS1* | 0.700 | 0.008 |
| *PCDHGA5 – ITGAV* | 0.584 | 0.046 |
| *PCDHGA5 – PCDH15* | -0.561 | 0.046 |
| *PCDHGA7 – LIMS1* | -0.679 | 0.021 |
| *PCDHGA7 – PCDH12* | -0.693 | 0.018 |
| *PCDHGA7 – PCDH17* | -0.642 | 0.033 |
| *PCDHB4 – ITGAE* | -0.731 | 0.007 |
| *PCDHGA2 – ICAM1* | 0.675 | 0.032 |
| *PCDHGA2 – ITGAE* | -0.632 | 0.049 |
| *PCDHGA2 – FHOD1* | 0.774 | 0.005 |
| *PCDHB6 – ITGA1* | -0.577 | 0.049 |
| *ICAM1 – VCAM1* | 0.739 | 0.004 |
| *SELP – LIMS1* | -0.731 | 0.005 |
| *SELP – ITGA6* | -0.686 | 0.010 |
| *VCL – ITGA1* | -0.708 | 0.007 |
| *VCL – ITGA6* | -0.563 | 0.045 |
| *VCL – PCDH12* | -0.682 | 0.015 |
| *VCL – PCDH17* | -0.624 | 0.023 |
| *VCL – JAM2* | -0.723 | 0.005 |
| *LIMS1– ITGAV* | 0.712 | 0.009 |
| *LIMS1– ITGA1* | 0.625 | 0.022 |
| *LIMS1– ITGA6* | 0.695 | 0.008 |
| *ITGA1– ITGA6* | 0.683 | 0.010 |
| *ITGAV – FHOD1* | -0.674 | 0.016 |
| *ITGAV – JAM2* | 0.714 | 0.009 |
| *ITGAV – SELP* | -0.652 | 0.021 |
| *ITGA1 – PCDHGA7* | -0.692 | 0.018 |
| *ITGA1 – ITGAV* | 0.652 | 0.021 |
| *ITGA1 – JAM2* | 0.768 | 0.002 |
| *ITGA6 – JAM2* | 0.695 | 0.008 |
| *ITGA6 – PCDHGA7* | -0.791 | 0.004 |
| *ITGA6 – PCDH17* | 0.800 | 0.001 |
| *ITGA6 – ITGAV* | 0.670 | 0.017 |
