# Supplementary material for: New Cell Adhesion Molecules in Human Ischemic Cardiomyopathy. PCDHGA3 Implications in Decreased Stroke Volume and Ventricular Dysfunction
Source: PLoS One. 2016 Jul 29;11(7):e0160168. doi: 10.1371/journal.pone.0160168 (PMC4966940; doi:10.1371/journal.pone.0160168)
Supplement: S2 Table — (DOCX) [file pone.0160168.s002.docx]

**S2 Table. Relationships between the differentially expressed genes and hemodynamic and LV function parameters of ICM patients.**

| **Gene symbol** | **Ejection fraction** | **Fractional shortening** | **LVESD** | **LVEDD** | **Stroke volume** |
| --- | --- | --- | --- | --- | --- |
| *CADM1* | *r* = -0.534  *P* = 0.091 | *r* = -0.553  *P* = 0.078 | *r* = 0.517  *P* = 0.103 | *r* = 0.460  *P* = 0.154 | *r* = -0.340  *P* = 0.336 |
| *CDH6* | *r* = -0.417  *P* = 0.202 | *r* = -0.368  *P* = 0.266 | *r* = -0.190  *P* = 0.575 | *r* = -0.289  *P* = 0.388 | *r* = -0.058  *P* = 0.874 |
| *CDH13* | *r* = 0.283  *P* = 0.399 | *r* = 0.368  *P* = 0.265 | *r* = -0.171  *P* = 0.615 | *r* = -0.096  *P* = 0.778 | *r* = 0.526  *P* = 0.116 |
| *CDH26* | *r* = -0.055  *P* = 0.876 | *r* = -0.161  *P* = 0.637 | *r* = -0.014  *P* = 0.967 | *r* = -0.061  *P* = 0.859 | *r* = -0.353  *P* = 0.318 |
| *CLDN9* | *r* = -0.591  *P* = 0.072 | *r* = -0.609  *P* = 0.062 | *r* = 0.328  *P* = 0.354 | *r* = 0.258  *P* = 0.473 | *r* = -0.512  *P* = 0.159 |
| *CLDN12* | *r* = 0.027  *P* = 0.936 | *r* = 0.034  *P* = 0.922 | *r* = 0.275  *P* = 0.414 | *r* = 0.323  *P* = 0.332 | *r* = 0.149  *P* = 0.681 |
| *CTNNA3* | *r* = -0.028  *P* = 0.936 | *r* = -0.057  *P* = 0.869 | *r* = -0.092  *P* = 0.788 | *r* = -0.118  *P* = 0.729 | *r* = -0.195  *P* = 0.589 |
| *CTNND2* | *r* = -0.515  *P* = 0.102 | *r* = -0.412  *P* = 0.209 | *r* = 0.181  *P* = 0.595 | *r* = 0.088  *P* = 0.798 | *r* = -0.316  *P* = 0.374 |
| *DSC2* | *r* = -0.206  *P* = 0.544 | *r* = -0.211  *P* = 0.533 | *r* = 0.306  *P* = 0.360 | *r* = 0.292  *P* = 0.383 | *r* = -0.074  *P* = 0.838 |
| *DSP* | *r* = 0.090  *P* = 0.792 | *r* = 0.100  *P* = 0.769 | *r* = -0.316  *P* = 0.344 | *r* = -0.320  *P* = 0.337 | *r* = -0.296  *P* = 0.407 |
| *FHOD1* | *r* = 0.198  *P* = 0.559 | *r* = 0.165  *P* = 0.629 | *r* = 0.044  *P* = 0.899 | *r* = 0.082  *P* = 0.811 | *r* = -0.110  *P* = 0.763 |
| *GJA3* | *r* = -0.117  *P* = 0.732 | *r* = -0.100  *P* = 0.769 | *r* = 0.113  *P* = 0.741 | *r* = 0.114  *P* = 0.738 | *r* = 0.015  *P* = 0.967 |
| *GJC1* | *r* = 0.097  *P* = 0.778 | *r* = 0.111  *P* = 0.745 | *r* = 0.283  *P* = 0.398 | *r* = 0.350  *P* = 0.292 | *r* = 0.011  *P* = 0.976 |
| *ITGA1* | *r* = 0.059  *P* = 0.863 | *r* = 0.122  *P* = 0.721 | *r* = -0.473  *P* = 0.142 | *r* = -0.497  *P* = 0.120 | *r* = -0.409  *P* = 0.240 |
| *ITGA6* | *r* = 0.231  *P* = 0.495 | *r* = 0.316  *P* = 0.344 | *r* = -0.429  *P* = 0.188 | *r* = -0.398  *P* = 0.226 | *r* = 0.554  *P* = 0.096 |
| *ITGA9* | *r* = 0.372  *P* = 0.260 | *r* = 0.388  *P* = 0.239 | *r* = -0.168  *P* = 0.621 | *r* = -0.088  *P* = 0.797 | *r* = 0.625  *P* = 0.053 |
| *ITGAE* | *r* = -0.199  *P* = 0.558 | *r* = -0.177  *P* = 0.602 | *r* = 0.048  *P* = 0.888 | *r* = 0.015  *P* = 0.966 | *r* = 0.025  *P* = 0.946 |
| *ITGAM* | *r* = -0.491  *P* = 0.150 | *r* = -0.458  *P* = 0.183 | *r* = 0.066  *P* = 0.856 | *r* = -0.025  *P* = 0.945 | *r* = -0.457  *P* = 0.216 |
| *ITGAV* | *r* = 0.292  *P* = 0.414 | *r* = 0.338  *P* = 0.340 | *r* = -0.348  *P* = 0.325 | *r* = -0.311  *P* = 0.381 | *r* = 0.523  *P* = 0.148 |
| *JAM2* | *r* = 0.260  *P* = 0.440 | *r* = 0.311  *P* = 0.352 | *r* = -0.548  *P* = 0.081 | *r* = -0.541  *P* = 0.086 | *r* = 0.544  *P* = 0.104 |
| *JUP* | *r* = 0.109  *P* = 0.750 | *r* = 0.144  *P* = 0.672 | *r* = -0.049  *P* = 0.885 | *r* = -0.023  *P* = 0.945 | *r* = 0.101  *P* = 0.782 |
| *LIMS1* | *r* = 0.130  *P* = 0.704 | *r* = 0.200  *P* = 0.556 | *r* = 0.020  *P* = 0.954 | *r* = 0.074  *P* = 0.830 | *r* = 0.358  *P* = 0.309 |
| *PCDH7* | *r* = 0.031  *P* = 0.932 | *r* = 0.023  *P* = 0.950 | *r* = -0.274  *P* = 0.444 | *r* = -0.287  *P* = 0.422 | *r* = 0.246  *P* = 0.524 |
| *PCDH12* | *r* = 0.176  *P* = 0.627 | *r* = 0.234  *P* = 0.516 | *r* = -0.471  *P* = 0.169 | *r* = -0.453  *P* = 0.188 | *r* = 0.542  *P* = 0.132 |
| *PCDH15* | *r* = 0.112  *P* = 0.744 | *r* = 0.033  *P* = 0.924 | *r* = 0.083  *P* = 0.809 | *r* = 0.088  *P* = 0.796 | *r* = -0.121  *P* = 0.738 |
| *PCDHB4* | *r* = 0.333  *P* = 0.347 | *r* = 0.383  *P* = 0.275 | *r* = -0.335  *P* = 0.344 | *r* = -0.277  *P* = 0.439 | *r* = 0.361  *P* = 0.340 |
| *PCDHB6* | *r* = 0.258  *P* = 0.461 | *r* = 0.284  *P* = 0.453 | *r* = -0.266  *P* = 0.457 | *r* = -0.128  *P* = 0.724 | *r* = 0.374  *P* = 0.321 |
| *PCDHB10* | *r* = 0.304  *P* = 0.426 | *r* = 0.334  *P* = 0.380 | *r* = -0.159  *P* = 0.682 | *r* = -0.076  *P* = 0.847 | *r* = 0.400  *P* = 0.326 |
| *PCDHB15* | *r* = -0.068  *P* = 0.843 | *r* = -0.191  *P* = 0.574 | *r* = 0.534  *P* = 0.056 | *r* = 0.541  *P* = 0.052 | *r* = -0.454  *P* = 0.188 |
| *PCDHGA2* | *r* = -0.307  *P* = 0.422 | *r* = -0.278  *P* = 0.469 | *r* = 0.443  *P* = 0.232 | *r* = 0.436  *P* = 0.241 | *r* = -0.435  *P* = 0.282 |
| *PCDHGA3* | *r* = -0.793  *P* = 0.004 | *r* = -0.822  *P* = 0.002 | *r* = 0.867  *P* = 0.001 | *r* = 0.781  *P* = 0.005 | *r* = -0.826  *P* = 0.003 |
| *PCDHGA5* | *r* = 0.160  *P* = 0.638 | *r* = 0.197  *P* = 0.562 | *r* = 0.182  *P* = 0.591 | *r* = 0.268  *P* = 0.426 | *r* = 0.321  *P* = 0.366 |
| *PCDHGA7* | *r* = 0.361  *P* = 0.340 | *r* = 0.284  *P* = 0.459 | *r* = -0.161  *P* = 0.679 | *r* = -0.114  *P* = 0.771 | *r* = -0.100  *P* = 0.813 |
| *PCDHGB3* | *r* = -0.448  *P* = 0.194 | *r* = -0.503  *P* = 0.138 | *r* = 0.609  *P* = 0.062 | *r* = 0.563  *P* = 0.090 | *r* = -0.579  *P* = 0.102 |
| *PCDHGB7* | *r* = 0.265  *P* = 0.459 | *r* = 0.309  *P* = 0.384 | *r* = -0.538  *P* = 0.109 | *r* = -0.539  *P* = 0.108 | *r* = 0.146  *P* = 0.708 |
| *PCDHGC3* | *r* = 0.065  *P* = 0.868 | *r* = 0.053  *P* = 0.893 | *r* = 0.202  *P* = 0.602 | *r* = 0.255  *P* = 0.508 | *r* = 0.421  *P* = 0.299 |
| *PKP2* | *r* = -0.025  *P* = 0.941 | *r* = 0.091  *P* = 0.790 | *r* = -0.176  *P* = 0.606 | *r* = -0.191  *P* = 0.574 | *r* = 0.312  *P* = 0.379 |
| *PKP4* | *r* = -0.055  *P* = 0.873 | *r* = -0.108  *P* = 0.752 | *r* = 0.322  *P* = 0.334 | *r* = 0.325  *P* = 0.329 | *r* = -0.179  *P* = 0.621 |
| *PVRL3* | *r* = 0.013  *P* = 0.972 | *r* = 0.056  *P* = 0.878 | *r* = -0.067  *P* = 0.854 | *r* = -0.038  *P* = 0.916 | *r* = 0.484  *P* = 0.187 |
| *SELP* | *r* = -0.304  *P* = 0.363 | *r* = -0.373  *P* = 0.259 | *r* = 0.146  *P* = 0.668 | *r* = 0.076  *P* = 0.825 | *r* = -0.549  *P* = 0.100 |
| *VCL* | *r* = 0.022  *P* = 0.949 | *r* = 0.013  *P* = 0.971 | *r* = 0.409  *P* = 0.211 | *r* = 0.456  *P* = 0.159 | *r* = -0.091  *P* = 0.803 |
| *VCAM1* | *r* = -0.379  *P* = 0.251 | *r* = -0.390  *P* = 0.236 | *r* = 0.008  *P* = 0.981 | *r* = -0.065  *P* = 0.849 | *r* = -0.238  *P* = 0.507 |

LV, left ventricular; ICM, ischemic cardiomyopathy; LVESD, left ventricular end-systolic diameter; LVEDD, left ventricular end-diastolic diameter.
